# Intergenerational transmission of stress: Multi-domain stressors from maternal childhood and pregnancy predict children’s mental health in a racially and socioeconomically diverse, multi-site cohort

**DOI:** 10.1007/s00127-022-02401-z

**Published:** 2023-02-03

**Authors:** Nicole R. Bush, Amanda Noroña-Zhou, Michael Coccia, Kristen L. Rudd, Shaikh I. Ahmad, Christine T. Loftus, Shanna H. Swan, Ruby H. N. Nguyen, Emily S. Barrett, Frances A. Tylavsky, W. Alex Mason, Catherine J. Karr, Sheela Sathyanarayana, Kaja Z. LeWinn

**Affiliations:** 1 Department of Psychiatry and Behavioral Sciences, San Francisco (UCSF), Weill Institute for Neurosciences, University of California, Box 0110, 550 16th Street, CA 94143 San Francisco, USA; 2 Department of Pediatrics, UCSF, San Francisco, CA USA; 3 Center for Health and Community, Division of Developmental Medicine UCSF, San Francisco, CA USA; 4 Department of Occupational and Environmental Health Sciences, University of Washington, Seattle, WA USA; 5 Department of Environmental Medicine and Public Health, Icahn School of Medicine at Mount Sinai, New York, NY USA; 6 Department of Epidemiology and Community Health, University of Minnesota, Minneapolis, MN USA; 7 Department of Biostatistics and Epidemiology, Rutgers School of Public Health, Piscataway, NJ USA; 8 Department of Preventive Medicine, University of Tennessee Health Sciences Center, Memphis, TN USA; 9 Department of Pediatrics, University of Washington, Seattle, WA USA; 10 Seattle Children’s Research Institute, Seattle, WA USA

**Keywords:** Child mental health, Childhood trauma, Pregnancy stress, Intergenerational transmission, Child psychopathology

## Abstract

**Purpose:**

Despite growing recognition that unfortunately common maternal stress exposures in childhood and pregnancy may have intergenerational impacts on children’s psychiatric health, studies rarely take a life course approach. With child psychopathology on the rise, the identification of modifiable risk factors is needed to promote maternal and child well-being. In this study, we examined associations of maternal exposure to childhood traumatic events (CTE) and pregnancy stressful life events (PSLE) with child mental health problems in a large, sociodemographically diverse sample.

**Methods:**

Participants were mother–child dyads in the ECHO-PATHWAYS consortium’s harmonized data across three U.S. pregnancy cohorts. Women completed questionnaires regarding their own exposure to CTE and PSLE, and their 4–6-year-old child’s mental health problems using the Child Behavior Checklist (CBCL). Regression analyses estimated associations between stressors and child total behavior problems, adjusting for confounders.

**Results:**

Among 1948 dyads (child age *M* = 5.13 (SD = 1.02) years; 38% Black, 44% White; 8.5% Hispanic), maternal history of CTE and PSLE were independently associated with children’s psychopathology: higher CTE and PSLE counts were related to higher total problems ([ß_CTE_ = 0.11, 95% CI [.06, .16]; ß_SLE_ = 0.21, 95% CI [.14, 0.27]) and greater odds of clinical levels of problems (OR_CTE_ = 1.41; 95% CI [1.12, 1.78]; OR_PSLE_ = 1.36; 95% CI [1.23, 1.51]). Tests of interaction showed PSLEs were more strongly associated with child problems for each additional CTE experienced.

**Conclusion:**

Findings confirm that maternal exposure to CTE and PSLE are independently associated with child mental health, and history of CTE exacerbates the risk associated with PSLE, highlighting intergenerational risk pathways for early psychopathology. Given the prevalence of these exposures, prevention and intervention programs that reduce childhood trauma and stress during pregnancy will likely positively impact women’s and their children’s health.

**Supplementary Information:**

The online version contains supplementary material available at 10.1007/s00127-022-02401-z.

Rising rates of children’s psychiatric problems present a significant public health concern [1]. The etiology of children’s mental health is complex, with salient risk factors occurring across time and at multiple levels of influence, potentially with intergenerational underpinnings [2]. The Developmental Origins of Health and Disease (DOHaD) framework posits that maternal experiences of stress during pregnancy influence children’s developmental outcomes, including mental health [3], through both maternal-placental-fetal biological pathways and postnatal environmental differences [4–9]. Fetal development is rapid and particularly sensitive to intrauterine inputs (e.g. maternal immune system activity and circulating stress hormone levels), in an effort to respond adaptively to maternal input to maximize survival [10], yet this can have lifelong implications for offspring health across the life course [11], and the high rates of women’s exposure to violence and adversity during pregnancy [12] suggest implications for population health. Empirical work conducted primarily in small samples provides support for this concern; women who experience more types of stressful life events during pregnancy are more likely to have infants with a range of problems such as lower self-regulation, lower cognitive performance, more fearfulness, and more behavior problems [13–15]. Accumulating evidence suggests that these associations may persist through early childhood in the form of behavior problems [16, 17] and into adolescence in the form of internalizing and externalizing mental health problems [18–20].

A newer line of DOHaD inquiry, built upon findings from non-human animal models, includes preconception maternal experiences as important predictors of offspring development and health [21]. For example, a woman’s exposure to traumatic events during her own childhood not only is a salient predictor of her own health and wellbeing but also may have lingering effects on her child’s development, such as an increased risk for early birth [22] and for psychopathology [23, 24]. Although exposure rates of adversity in childhood are common in women—several epidemiological studies have shown that more than half of adult women report exposure to childhood adversity [25–27]—effects of traumatic childhood experiences on a woman’s subsequent offspring are not well understood, and the emphasis on prenatal exposures transmitting risk to offspring has likely obscured understanding of the intergenerational impact of earlier stressors. Indeed, both childhood and pregnancy may be sensitive periods for women during which stress exposure may affect her body and behavior in a manner relevant to her child’s development and health, yet empirical attention to these maternal sensitive period histories of adversity and children’s mental health is lacking. Evidence from a modest sample with deep phenotyping showed that mothers’ exposures to adversity during pregnancy and childhood uniquely predicted maternal immune responses [28], which have relevance for fetal gestational environments. In a larger cohort study, childhood traumatic events, but not pregnancy events, predicted maternal pregnancy hormones related to birth timing and offspring neurodevelopmental outcomes [29]. Another study found that maternal mental health during the perinatal period mediated maternal childhood trauma effects on childhood internalizing and externalizing problems [30]. Although data are limited, it follows that simultaneous consideration of stressors experienced across multiple key developmental periods in the mother’s lifespan (e.g., early childhood and pregnancy) [9] may better account for the intergenerational impact of a mother’s stressful experiences on her child’s mental health. Such a life course perspective has the potential to illuminate prenatal and early postnatal family-focused intervention targets to help curb the rising rates of children’s mental health problems.

Existing studies, however, are limited in a number of ways, and several important questions remain. First, very few prior studies have examined women’s exposure to both childhood trauma and major pregnancy stressors to evaluate the additive risk of stressful experiences across multiple life course windows in relation to children’s psychiatric outcomes. Second, most study samples are small, and the few large-scale studies of associations between maternal stress exposure and child development harness samples of predominantly White, middle-to-upper-income individuals who do not accurately reflect the diversity and range of living conditions within the United States [18, 19]. Differences in caregiver and family social policies such as care leave benefits differ substantially across countries [31, 32], therefore, extant findings may not generalize broadly. Moreover, families of color or with strained socioeconomic resources are disproportionately exposed to a variety of stressors [12], thus stress-focused research should include communities with higher exposures. While DOHaD processes are not likely to function differently in underrepresented populations, there remains a need to study these processes in more diverse samples to improve inclusion and enhance understanding of these phenomena in populations for which policies are being developed. Studies that recruit more diverse samples often have significantly smaller sample sizes, resulting in limited power and concerns about reproducibility, or do not adequately adjust for potential confounders. Thus, the impact of stressful experiences may be best evaluated in large, diverse samples.

The current study sought to address these gaps by utilizing harmonized data from the ECHO-PATHWAYS consortium (https://deohs.washington.edu/echo), which combines three cohorts drawn from six recruitment regions across the US. The primary aim was to examine whether maternal experiences of stressful events during pregnancy and traumatic events during her own childhood, are independently associated with children’s mental health (emotional and behavioral) problems at age 4–6. This is one of the first studies to use a large, multi-cohort sample of sociodemographically diverse dyads across various U.S. geographic regions to examine the intergenerational transmission of maternal stress effects on children’s mental health. In addition to evaluating multiple types of stressors from two sensitive periods of women’s lives, our study also advances the literature through rigorous adjustment for pre- and postnatal confounding variables, in a staged, multi-model approach that considers how many potential confounders may actually lie on the causal path. Such adjustment allows specific examination of maternal stress exposures as predictors of child psychopathology, above and beyond well-established correlates of maternal stress and/or child psychopathology, such as birth outcomes and postnatal maternal mental health at the outcome timepoint. We hypothesized that stressful events during pregnancy and maternal childhood trauma would independently predict higher levels of child problems, indicating an accumulating impact of stressors across the maternal life course. As the biological sex of the developing fetus may modify the effects of maternal prenatal stress on child development [33], we also examined whether child sex modified the associations of interest. Finally, in post-hoc analyses, we examined whether a maternal history of childhood trauma would modify the association between pregnancy stressors and child outcomes, to explore the possibility that the two maternal exposures interact synergistically to exacerbate risk for adverse child outcomes.

## Methods

### Participants

Participants were 1948 mother–child dyads with outcome data who were enrolled in one of three prospective prenatal cohorts participating in the U.S.-based NIH ECHO-PATHWAYS consortium [34]: The Infant Development and the Environment Study (TIDES), the Conditions Affecting Neurocognitive Development and Learning in Early childhood (CANDLE) study, and the Global Alliance to Prevent Prematurity and Stillbirth (GAPPS) study.

The TIDES study recruited women older than 18 years with a healthy pregnancy from four university-based prenatal clinics: the University of San Francisco, California (San Francisco, CA), the University of Rochester Medical Center (Rochester, NY), the University of Minnesota (Minneapolis, MN), and the University of Washington/Seattle Children’s (Seattle, WA; [35]. In the CANDLE study, based at the University of Tennessee Health Science Center (Memphis, TN), healthy women between 16 and 40 years of age with low medical risk and plans to deliver at a participating study hospital were enrolled in their second trimester of pregnancy using community-based and clinic-based recruitment [34]. The GAPPS study enrolled pregnant women over the age of 18, attending select prenatal clinics in the Seattle and Yakima, WA areas (www.gapps.org). All three of these cohorts included research visits during pregnancy, a birth exam and/or birth record review, and a postnatal visit around the child age 4–6 years. Extant and prospectively collected individual-level data were pooled and harmonized across cohorts with enrollment into ECHO-PATHWAYS. All mothers provided consent for themselves and their children. All ECHO-PATHWAYS research activities were approved by the University of Washington IRB and relevant partner institutions and have therefore been performed in accordance with the ethical standards laid down in the 1964 Declaration of Helsinki and its later amendments.

### Measures

*Maternal childhood trauma exposure (CTE)*. Exposure to trauma during childhood was assessed retrospectively during the pregnancy period via a maternal report on three items from the Traumatic Life Events Questionnaire, Version 2 (TLEQ 2; [36] that were asked within each of the three cohorts. Participants reported whether they were exposed to physical abuse and witnessing family violence before age 18, and sexual abuse before age 13. A count of the number of types of trauma experienced during childhood was created (range = 0–3).

*Pregnancy stressful life events (PSLE)*. Women reported retrospectively on the number of types of stressful life events they experienced during pregnancy using a list of 14 events adapted from the Centers for Disease Control and Prevention Pregnancy Risk Assessment Monitoring System (PRAMS) survey [37]. During a postnatal follow-up assessment (age 4–6 for GAPPS, age 6 for TIDES, age 8 for CANDLE), participants endorsed or declined statements about experiences with illness, death, relationship problems, housing difficulties, legal issues, and financial problems they had faced during the index pregnancy. Affirmative responses were summed (range = 0–14). Evaluations of retrospective reports have established their validity and robustness to bias [38, 39], supporting this measurement timing.

*Child mental health*. The Child Behavior Checklist (CBCL; [40] is a caregiver-report form used to assess a wide range of emotional and behavior problems in children and adolescents. At the age 4–6 visit in CANDLE and GAPPS and the age 6 visit in TIDES, mothers reported on each symptom/behavior as ‘not true’ (0), ‘sometimes/somewhat true’ (1), or ‘very/often true’ (2) of their child. Cohorts administered the age-appropriate version of the form (see supplemental text for details). The primary outcome variable, the Total Problems raw score, was calculated as sum of all symptoms/behaviors related to depression, anxiety, aggression, inattention, and hyperactivity, among others. To aid the interpretation of clinically meaningful effects, two dichotomous indicators were also created to represent CBCL-form-specific normalized Total Problems scores at-or-above Borderline (84th percentile) and Clinical (90th percentile) thresholds, per manual [40].

*Covariates*. The ECHO-PATHWAYS cohorts are well-characterized, allowing for robust adjustment of potential confounders, determined a priori and including: study site; version of outcome measure (two forms were utilized); family income adjusted for household size and region; maternal parity, pre-pregnancy BMI, education, age, prenatal smoking, and postnatal depression; parent-reported child race/ethnicity,^1^ sex, gestational age at birth, birth weight, breastfeeding history, and age at the outcome. Covariates were considered for inclusion within staged models, based on their potential roles as confounders as well as concerns about their potential for being on the causal pathway (i.e., serving as mediators) between the exposure and child outcome (see data analysis details below). See Supplemental text for additional details on measurement and selection of covariates.

### Data analysis

Primary analyses examined the association between maternal stress exposures (CTE and PSLE) and child mental health in the multi-cohort sample using multiple linear regression to predict child Total Problems sum scores. Based on the literature, and to consider which variables were potentially, or likely to be, on the causal path, we developed a staged adjustment approach, allowing exploration of the influence of increasing levels of adjustment on results. First, associations were examined within a “minimally-adjusted” model (Model 1), controlling for data-collection site and CBCL form. In the second model (Model 2), which we defined as the “fully-adjusted” or primary results model, a set of variables identified as major potential confounders were included as covariates: log-transformed family income adjusted for household size and region (assessed at outcome timepoint); maternal parity, pre-pregnancy BMI, education, and age at enrollment; and child race/ethnicity, sex, and age at the outcome. In the third “extended” model (Model 3), analyses additionally included variables that may be potential confounders but may also be on the mechanistic or causal path between stress exposures and child mental health, including: prenatal smoking, child gestational age at birth, child birth weight, breastfeeding history, and concurrent maternal self-report of depression. As this third model was likely to attenuate results toward the null, due to covarying for effects likely “on the path”, its results were considered informative but secondary. All non-categorical covariates were mean-centered. (See supplement for additional details regarding models and covariates.)

Using this same covariate adjustment strategy, two logistic regression models were used to examine the stress exposures and their associations with (1) borderline child mental health threshold scores and (2) clinically significant threshold scores. Effect modification by child biological sex was examined using cross-product interaction terms between each stressor and sex, entered together, in fully adjusted models. Finally, post-hoc analyses examined the cross-product interaction term between the two maternal exposures, and sensitivity analyses examined the main-effects, fully adjusted, and extended models separately within each cohort.

The MICE (Multivariate Imputation by Chained Equations) package in R was used to impute missing values for all predictors and covariates across the three-cohort sample. Across both primary exposures and covariates, missing data ranged from 0.2 to 13% (Table 1). The imputation model used all other variables as predictors for each incomplete data variable, including sex interaction terms. As a sensitivity analysis, fully adjusted primary models were tested within a complete-case dataset.

**Table 1:** Details of the study sample and primary study variables, by cohort

Characteristic^1^	*N* = 1948	CANDLE, *N* = 1030	GAPPS, *N* = 377	TIDES, *N* = 541
Recruitment site				
Memphis	1030 (53%)	1030 (100%)	0 (0%)	0 (0%)
SF	138 (7.1%)	0 (0%)	0 (0%)	138 (26%)
UMN	153 (7.9%)	0 (0%)	0 (0%)	153 (28%)
Rochester	133 (6.8%)	0 (0%)	0 (0%)	133 (25%)
Seattle (TIDES)	117 (6.0%)	0 (0%)	0 (0%)	117 (22%)
Seattle (GAPPS)	204 (10%)	0 (0%)	204 (54%)	0 (0%)
Yakima	173 (8.9%)	0 (0%)	173 (46%)	0 (0%)
CBCL form version				
Age 1–5 form	1306 (67%)	1030 (100%)	276 (73%)	0 (0%)
Age 6–18 form	642 (33%)	0 (0%)	101 (27%)	541 (100%)
Child biological sex				
Female	987 (51%)	517 (50%)	185 (49%)	285 (53%)
Male	957 (49%)	513 (50%)	192 (51%)	252 (47%)
Missing	4	0	0	4
Child age at outcome (years)	5.13 (± 1.02)	4.31 (± 0.38)	5.62 (± 0.76)	6.34 (± 0.37)
Missing	39	27	0	12
Child race/Ethnicity				
Black (alone; non-Hispanic)	719 (38%)	656 (65%)	9 (2.4%)	54 (10%)
White (alone; non-Hispanic)	831 (44%)	266 (26%)	234 (62%)	331 (64%)
Other (and multiple race; non-Hispanic)	189 (9.9%)	51 (5.1%)	60 (16%)	78 (15%)
Hispanic (any race)	161 (8.5%)	32 (3.2%)	73 (19%)	56 (11%)
Missing	48	25	1	22
Maternal age (years)	34 (± 6)	31 (± 6)	37 (± 6)	38 (± 5)
Missing	37	27	4	6
Maternal pre-pregnancy BMI	27 (± 7)	28 (± 8)	27 (± 7)	26 (± 6)
Missing	40	3	24	13
Maternal education				
< high school	76 (3.9%)	54 (5.3%)	3 (0.8%)	19 (3.6%)
High school diploma or GED	472 (25%)	404 (39%)	33 (8.9%)	35 (6.6%)
Technical school	330 (17%)	134 (13%)	109 (30%)	87 (16%)
College degree	537 (28%)	258 (25%)	124 (34%)	155 (29%)
Graduate or professional degree	510 (26%)	173 (17%)	100 (27%)	237 (44%)
Missing	23	7	8	8
Gravidity	2.00 [1.00, 3.00]	2.00 [1.00, 3.00]	2.00 [1.00, 3.00]	2.00 [1.00, 3.00]
Missing	29	0	12	17
Annual household income (regionally and-inflation-adjusted $)	55,751 [23,863, 86,768]	31,818 [13,557, 64,281]	85,984 [49,133, 122,834]	101,749 [48,839, 155,382]
Missing	99	70	6	23
PSLE (sum 0–14)	1.51 (± 1.77)	1.69 (± 1.93)	1.42 (± 1.60)	1.30 (± 1.59)
Missing	262	238	7	17
CTE (sum 0–3)	0.52 (± 0.80)	0.52 (± 0.78)	0.52 (± 0.85)	0.51 (± 0.81)
Missing	46	30	7	9
CBCL Child Mental Health Total Problems Sum Score	23 (± 18)	24 (± 19)	21 (± 18)	22 (± 17)
Child Mental Health at-or-above Borderline threshold	208 (11%)	92 (8.9%)	30 (8.0%)	86 (16%)
Child Mental Health at-or-above Clinical threshold	119 (6.1%)	51 (5.0%)	20 (5.3%)	48 (8.9%)

^1^Table reports *n *(%) for categorical and dichotomous features

Median [IQR] for income data; and Mean(± SD) for all other continuous and pseudo-continuous features

## Results

Table 1 provides the demographic characteristics of the current sample as well as the descriptive statistics for the exposures and outcomes used in analyses. Briefly, children (51% female) were 4–6 years old at the time of the assessment and represented diverse racial and ethnic backgrounds. Among the participants, 26% of mothers reported exposure to both PSLE and CTE, 36% PSLE only, 39% neither PSLE or CTE, and 9% CTE only. PSLE and CTE were weakly, positively associated (*r* = 0.25, *p* < 0.01). Using a simple negative binomial regression model with CTE as a 4-level factor, we observed increasing rates of PSLE endorsement for those reporting 1 (IRR = 1.6) or 2 (IRR = 2.0) or 3 (IRR = 2.5) CTE exposures, compared to those reporting no trauma on the 3-item scale. All IRR estimates were significant, *p*’s < 0.001. Correlations among key study variables are presented in Supplemental Table 1. Among the three cohorts that comprise PATHWAYS, a comparison of the maternal stress exposure measures revealed that CANDLE participants reported higher levels of PSLE than TIDES (*p* < 0.01) and GAPPS (*p* < 0.05). The three cohorts were not significantly different in regard to CTE.

**Fig. 1:**
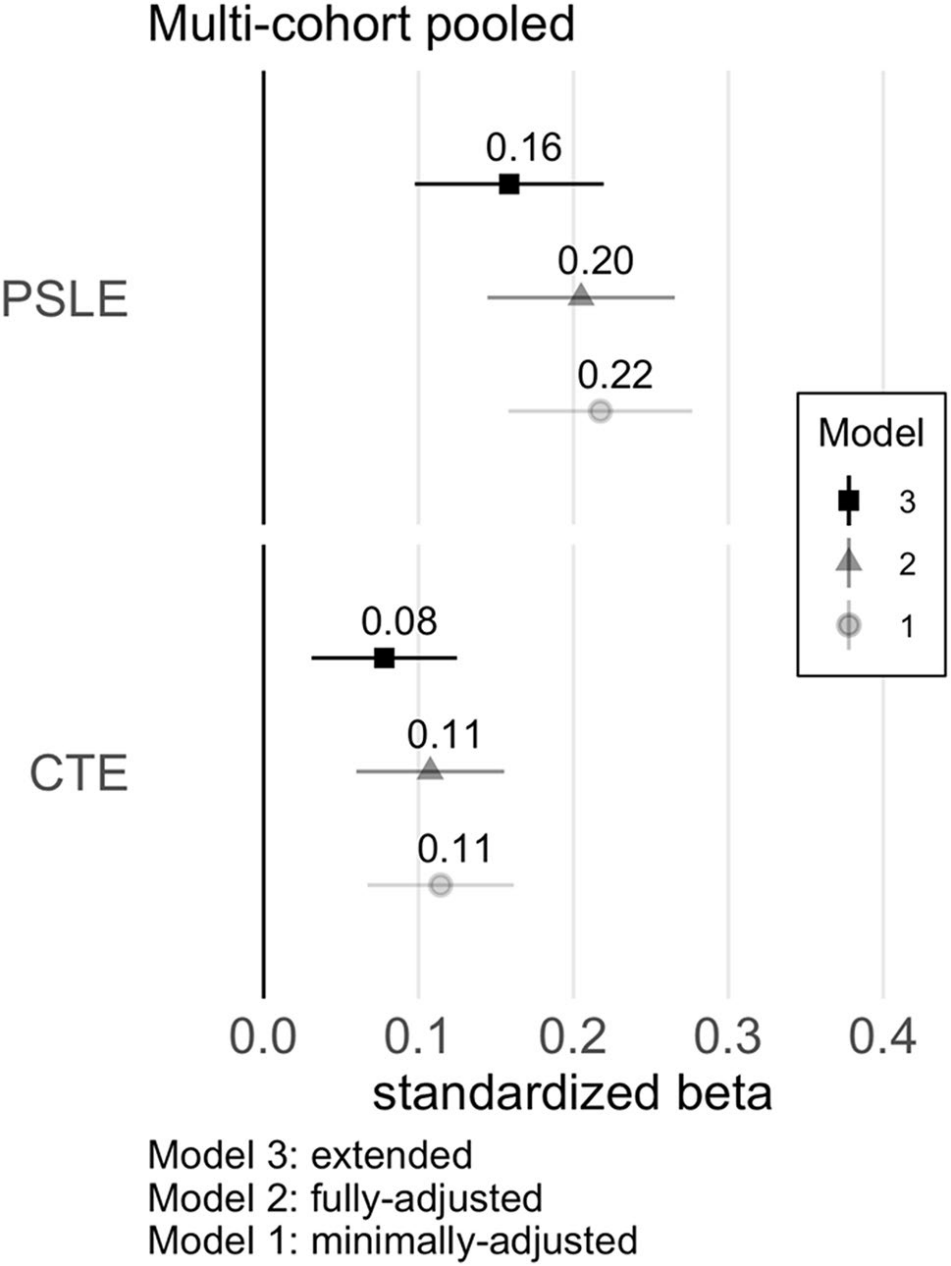
Illustration of Standardized Coefficients from Regression Models, pooled cohorts sample. Standardized estimates with 95% CI. Model 1 covaries for the data-collection site and CBCL form. Model 2 adjusts for child age, child biological sex, child race-ethnicity, gravidity, maternal age, maternal BMI, maternal education, regionally adjusted income-X-household size, and child cohort birth year. Model 3 includes additional adjustments for maternal depression, prenatal smoking, gestational age, birthweight, and breastfeeding

Minimally adjusted regression models (Model 1) indicated independent, positive associations between maternal CTE and PSLE with child behavior problems (Fig. 1). In the fully adjusted, primary model (Model 2), maternal CTE and PSLE continued to have significant independent positive associations with child mental health problems, though associations were slightly attenuated (Fig. 1; Table 2). Collectively, the two predictors accounted for 5.7% of the variance in child outcome. In the extended model that included adjustment for potential pathway variables (Model 3), offspring gestational age (birth timing) and concurrent maternal depression were significantly associated with child mental health (see Supplemental Table 1 for full regression results), and coefficient estimates for the two maternal stress exposure predictors were further attenuated but remained significant (Table 2). Tests for effect modification by child biological sex (Supplemental Table 2) were not significant for either CTE (*p* = 0.70) or PSLE (*p* = 0.30).

**Table 2:** Unstandardized coefficients from fully adjusted and extended regression models predicting child CBCL total problems raw score in ECHO-PATHWAYS participants

	MI-across-cohort		CANDLE		GAPPS		TIDES	
Model#	2	3	2	3	2	3	2	3
CTE sum	2.47*** (0.56)	1.79** (0.55)	2.18** (0.80)	1.45 + (0.78)	2.40* (1.19)	1.85 (1.20)	2.90** (0.96)	2.33* (0.95)
PSLE sum	2.12*** (0.30)	1.64*** (0.31)	1.69*** (0.42)	1.17** (0.42)	2.65*** (0.63)	2.52*** (0.64)	2.51*** (0.51)	1.88*** (0.52)
*N*	1948	1948	1030	1030	377	377	541	541
R^2^	0.13	0.18	0.11	0.19	0.20	0.23	0.19	0.23

*MI* multiple imputation, *CTE* childhood trauma exposure, *PSLE* pregnancy stressful life events

***p < 0.001; **p < 0.01; *p < 0.05; + *p* < 0.1

Model 2 adjusts for child age, child biological sex, child race-ethnicity, gravidity, maternal age, maternal BMI, maternal education, regionally adjusted income-X-household size, and child cohort birth year

Model 3 includes additional adjustments for maternal depression, prenatal smoking, gestational age, birthweight, and breastfeeding

Full table including all covariate coefficients is provided in the Supplement

In cohort-specific sensitivity analyses, conducted to ascertain whether associations were driven by a particular cohort, patterns of association were similar to those found in the pooled analyses (Supplemental Table 3). For example, for the primary model (2), CTE and PSLE were each significant predictors of child problems within each cohort. These results suggest that the pooled results were not driven by one particular cohort.

Fully adjusted logistic regression results from primary Model 2 (Table 3) showed that reported experience of a CTE or PSLE was related to a 41% (95% CI = 1.12, 1.78) or 36% (95% CI = 1.23, 1.51) increase, respectively, in the odds of child mental health problems occurring at or above the Clinical Problems threshold. For the Borderline threshold, reporting a CTE or PSLE was related to 34% (95% CI = 1.12, 1.62) or 23% (95% CI = 1.13, 1.35) respective increase in the odds of child mental health problems. Results for the extended model (Model 3) showed a decrease in the estimated coefficients (Table 3).

**Table 3:** Odds ratios for child mental health problems at borderline and clinical thresholds in ECHO-PATHWAYS participants

	CBCL Borderline Problems (84th %ile)		CBCL Clinical Problems (90th %ile)	
Model#	2	3	2	3
Variable	OR (95% CI)	OR (95% CI)	OR (95% CI)	OR 95% CI
CTE	1.34 (1.12, 1.62)	1.27 (1.04, 1.55)	1.42 (1.12, 1.78)	1.39 (1.09, 1.77)
PSLE	1.23 (1.13, 1.35)	1.21 (1.08, 1.30)	1.36 (1.23, 1.51)	1.33 (1.19, 1.48)

*OR* Odds Ratio, *CI* Confidence Interval, *CTE* (maternal) childhood trauma exposure; *PSLE* pregnancy stressful life events

Model 2 adjusts for child age, child biological sex, child race-ethnicity, gravidity, maternal age, maternal BMI, maternal education, regionally adjusted income-X-household size, and child cohort birth year

Model 3 includes additional adjustments for maternal depression, prenatal smoking, gestational age, birthweight, and breastfeeding

Full table including all covariate coefficients is provided in the Supplement

**Fig. 2:**
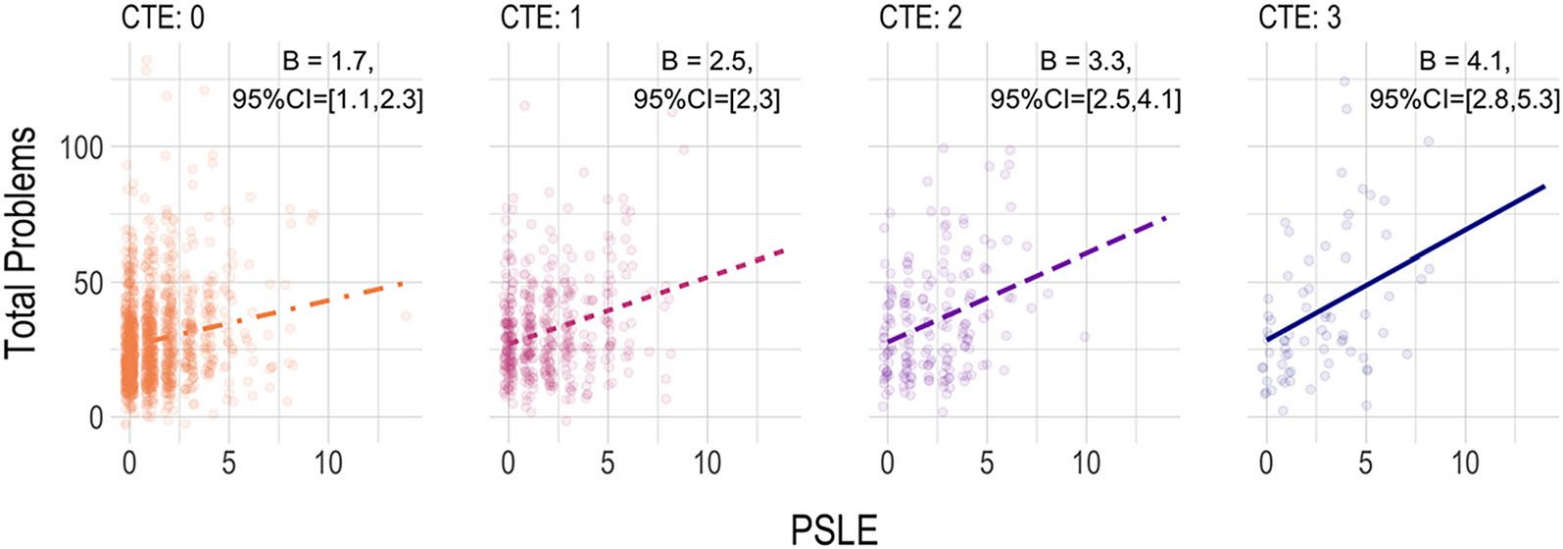
Illustration of the Interaction between PSLE and CTE predicting Child Total Problems. Plots of the Simple Slopes (unstandardized, with 95% CI) of the PSLE effect at CTE exposure levels, overlayed with partial residuals, shown from the first (of 10) imputed dataset. Plots present results from Model 2, which covaried for data-collection site and CBCL form, child age, child biological sex, child race-ethnicity, gravidity, maternal age, maternal BMI, maternal education, regionally adjusted income-X-household size, and child cohort birth year

In light of the pattern of significant prediction by both exposures, to ascertain the potential exacerbation of risk related to PSLE for those who experienced CTE, we conducted post-hoc tests of the interaction between CTE and PSLE, wherein the cross-products of the mean-centered predictor terms were added to Model 2 (primary model). Results showed a significant modification of the association between PSLE and child Total Problems by the maternal history of CTE (*B* = 0.78, 95% CI = 0.22, 1.3, *p* < 0.01). Probing the interaction by examining simple slopes at various levels of CTE exposure (see Fig. 2) revealed significant associations between PSLE and child Problems at all levels of CTE, but stronger associations at higher levels of CTE exposure, such that the simple slope between PSLE and Problems for women with a history of all 3 types of childhood trauma was nearly three times as large (*b* = 3.27) as the slope for women without any reported history of those exposures (*b* = 1.33).

Sensitivity analysis using complete cases (*N* = 1484) were consistent with the results of the imputed sample (see Supplemental Table 2).

## Discussion

Leveraging a large, multi-site U.S. cohort with socioeconomic, racial, and geographic diversity, this study supports our hypothesis that both maternal history of childhood trauma exposure (CTE) and exposure to major stressful life events during pregnancy (PSLE) predict children’s mental health at age 4–6 years. Notably, both exposures independently predicted greater odds of children having mental health problems at or above borderline or clinical thresholds, suggesting clinically meaningful impact of each exposure type. Moreover, the findings provide much needed evidence from families not typically represented in the current evidence base of cohorts that are predominantly comprised of White, middle-class individuals. Here we expand understanding of these associations to lower-income families and communities of color who are disproportionately exposed to adversity and disadvantage and who reside in the U.S., a country that lacks universal pre- and postnatal health care and leave benefits. Although effect sizes for each predictor were fairly small in our primary models (ßs = 0.11 for childhood and 0.21 for pregnancy), combined, they have a greater impact, and explained 5.7% of the variance in child problems. Moreover, maternal stressors interacted such that exposure to stressors in pregnancy were more strongly associated with child problems for women with histories of childhood trauma. In sum, the predictors in this interaction model explained 6.1% of the variance in problems. Such effects on early childhood behavioral functioning have the potential to contribute to cascading trajectories of problems and a sizable impact at the population level [42, 43]. Importantly, the consistency of the hypothesized associations found across and within the three unique cohorts suggest these associations can be expected in a range of populations, including in both higher and lower SES communities, across racial and ethnic groups, and across varied regions of the U.S.

Evidence for the independent contributions of CTE and PSLE supports the importance of considering maternal stress exposure across multiple sensitive periods of her life course [9] when attempting to understand the intergenerational transmission of stress to offspring health and wellbeing. Although child health research is increasingly considering the prenatal environment in etiological models, evidence points to both early childhood and pregnancy as periods of women’s development during which environments can shape her biology [28], health behaviors, and mental and physical health [44] outcomes with high relevance for her child’s subsequent fetal and postnatal development. The evidence for effect modification qualified those main effects, however, showing that although PSLE was significantly associated with child problems regardless of the level of CTE, the strength of the association increased for each additional type of childhood trauma mothers had experienced. For example, findings suggest that pregnancy stressors were associated with a nearly threefold increase in risk for child problems for women with a history of childhood sexual and physical violence and domestic violence (all 3 CTE), relative to women who reported experiencing none of those. This interaction finding suggests the potential for exposure to childhood trauma to affect maternal factors (likely some constellation of biology, behavior, and/or environment) in a manner that makes the maternal-child dyad more susceptible to the effects of perinatal stressor exposure.

Both prenatal programming of fetal development and postnatal rearing differences may account for associations found here. Indeed, although tests of mediation are beyond the scope of this paper, effects for both childhood and pregnancy stressors remained but were attenuated after adjustment of variables that may be either confounders or potentially on the mechanistic pathway. Accumulating evidence suggests that stress experienced across a woman’s lifetime can specifically affect birth timing through biological changes in maternal stress hormones and immune function [6, 29, 45]. In addition, there is longstanding evidence that histories of adversity and trauma affect adult mental health [44, 46], which is a strong predictor of childhood mental health [47], and one study found that maternal mental health mediated maternal childhood trauma effects on offspring mental health [30]. Accordingly, inclusion of maternal depression in our 3rd model likely attenuated those associations toward the null due to its likely role as a mediator, rather than improving precision by adjusting for confounding. Future studies should evaluate these and other likely pathways to support the identification of ideal time points and modifiable processes for intervention.

We found no evidence for moderation of the associations by child sex. As our sample was larger and more diverse than previously studied and included extensive confounder adjustment, this suggests that child sex may not be an effect modifier of maternal stress exposure and early childhood mental health. It may, however, be more relevant for other child outcomes or adolescents where pubertal timing and related hormonal changes play a stronger role in mental health development.

The findings from the sensitivity analyses within each of the three unique cohorts were largely consistent with the pooled results, showing effects were not driven by a particular cohort and increasing confidence that maternal exposure to stress can shape child psychiatric outcomes across populations. Inclusion of regionally, socioeconomically, and racially/ethnically diverse participants from these three cohorts improves confidence in the generalizability of effects and the likely value of universal prevention and intervention efforts in this domain. That said, the relatively smaller associations in the CANDLE cohort are worth reflection. Our data showed that exposure to childhood trauma and pregnancy stressors was largely consistent across the cohorts, although PSLE was slightly higher in CANDLE than in the other two cohorts. It is possible that those smaller estimated coefficients were due to higher exposure to other, unmeasured adversities prevalent in the Memphis community, including disproportionate exposure to neighborhood violent crime and other forms of structural racism, as well as unmeasured sources of family and community strengths, that may play a larger role in explaining child wellbeing in that population relative to the other cohorts studied here. To date, examinations of intergenerational stress exposures on child mental health outcomes in cohorts with a considerable representation of Southern U.S., Black, or low-income families have been limited, making comparisons difficult. Future research, conducted by scholars and community partners with expertise in these realms, should be prioritized [48].

The current results should be interpreted in the context of several limitations. First, maternal stress exposure during childhood and pregnancy were both assessed retrospectively. While a prospective collection of stress exposure is ideal, retrospective reports of significant life events (such as the experiences of severe illness, death of a close relative, or relationship changes) have been found to be valid and robust to recall bias over time [39, 49]. In addition, our two predictors were based on ascertainment of exposure (versus not) to major types of adverse events, an approach to assessing trauma that is feasible in large cohorts and tolerated fairly well by most research participants, but the richer assessment of stressor frequency, severity, or perceived impact may improve prediction of child health outcomes. A broader range of childhood trauma exposure types than that captured by our 3-item CTE scale might also have led to stronger associations. Further, all exposure and outcome measures included in the current analyses were completed by the participating mothers. While parent report of their child’s mental health is valid and essential in early childhood, confidence in the findings would be enhanced by clinical interview or observational data; however, this level of depth is difficult to achieve in an epidemiological cohort. To address one potential source of parent reporting bias (though see [50] for recent evidence that this bias is likely minimal), our final statistical model included adjustment for current maternal depression and other various maternal factors that may impact her report. Adjustment of maternal mood separately within the 3^rd^ model and finding the primary model was robust to its inclusion (though associations were slightly attenuated) is a significant strength, as it ruled out potential confounding while also acknowledging depression as a likely mediator of intergenerational transmission of stress. Furthermore, because our exposure measures captured slightly different multi-domain stressors (3 items of childhood traumatic events vs. 14 items of a range of pregnancy stressful life events), this study cannot be used to interpret relative effect sizes across maternal exposure developmental windows. Future research is needed to directly compare stressor exposure in these two life course periods, as well as assessment of total lifetime stress exposure of mothers from birth through pregnancy, which was not measured across the current cohorts. Such inclusion of total lifetime stressors, in addition to childhood and prenatal stressors, would illuminate the role of timing and sensitive periods for the intergenerational transmission of stress on health. Inclusion of additional sources of stress, especially systemic and structural inequities (e.g., residential segregation, institutional racism), with attention to disparities in which groups disproportionately experience those harms, is also critical for developing a comprehensive understanding of the role of stress exposure for developmental and health outcomes across generations.

In conclusion, this study finds a consistent, clinically meaningful pattern of association between maternal exposures to trauma and adversity during her own childhood and pregnancy and her child’s psychiatric symptoms around kindergarten age. Maternal stress exposure from both periods independently predicted children’s mental health, and interacted to compound risk, even after adjusting for a range of potential confounders, with evidence pointing to the possibility of both prenatal programming of fetal development and postnatal environmental pathways for the associations found. Accordingly, these findings highlight that prevention of trauma exposures, especially to children and pregnant women, should be a public health priority, not just for women’s wellbeing but for that of the next generation. Although screening efforts to identify individual histories of adversity are escalating [51], resources to address harms are limited and still in development, making prevention the more sound target. That said, many live with histories of harm, and there are effective solutions to improve outcomes for caregivers and their children. Interventions during pregnancy and the postnatal period have been shown to reduce stress for mothers [52, 53] and improve mental health outcomes in offspring [54], and the interaction findings from this study suggest that preventing or addressing pregnancy stress may be particularly important for women with histories of childhood trauma, whose children appear to have greater potential for exhibiting intergenerational transmission of harm. Moreover, aspects of the postnatal rearing environment, such as parental knowledge of child development [55] and positive parenting behaviors [56], have been shown to buffer children from the mental health risks associated with maternal trauma exposure histories. Harmonized measures of such protective factors were not available across the cohorts studied here; thus, the examination of resilience-enhancing factors should be a priority for future research. Moreover, children in this study were young, in a sensitive period of development during which emerging mental health problems can have cascading effects—it is important to determine the persistence of these associations and what factors offset risk of long-standing disease. Findings from this study show the importance of considering the accumulation of risks from adversity exposure across multiple periods of mothers’ lives and viewing child mental health from an intergenerational developmental perspective.

### Supplementary Information

Below is the link to the electronic supplementary material.

Supplementary file1 (DOCX 257 KB)

## Data Availability

Most ECHO-PATHWAYS data has been shared with the ECHO consortium and can be utilized for approved ECHO analysis proposals that leverage the U.S. NIH ECHO-wide Cohort data platform. Policies describing the use of ECHO data are available through the ECHO Coordinating Center, echocc@duke.edu. All of the specific data utilized for this published study may not publicly available but de-identified data may be available on request, subject to approval by the internal review board and under a formal data use agreement.

## Footnotes

1 1Race is a political and social construct that often serves as a proxy for the impact of racist practices and structural inequality. It is not a biological construct [41] and thus is included in analyses to capture unmeasured confounding with this premise in mind.
