# Supplementary material for: Intergenerational transmission of stress: Multi-domain stressors from maternal childhood and pregnancy predict children’s mental health in a racially and socioeconomically diverse, multi-site cohort
Source: Soc Psychiatry Psychiatr Epidemiol. 2023 Feb 3;58(11):1625–36. doi: 10.1007/s00127-022-02401-z (PMC10397362; doi:10.1007/s00127-022-02401-z)

**Supplemental Material for**

Intergenerational Transmission of Stress: Multi-domain Stressors from Maternal Childhood and Pregnancy Predict Children’s Mental Health in a Racially and Socioeconomically Diverse, Multi-site Cohort

**Supplemental Method**

**Child Behavior Checklist (CBCL) Form Versions**

As noted in the Method section, child mental health was assessed using the Child Behavior Checklist (CBCL). For each participant, one of two CBCL forms was administered, depending on the child’s age: the CBCL preschool form (ages 1.5-5 years) or the CBCL school age form (ages 6-18 years) [1] All CANDLE participants completed the preschool form, all TIDES participants completed the school-age forms, and GAPPS families completed a mix of both forms, given their broader age range at the study visit. The preschool form includes maternal report of the frequency of 99 child behaviors in the past two months, while the school age form includes report of 112 behaviors in the past six months. Caregivers rate these items on a scale of *Not True (0)*, *Somewhat or Sometimes True (1)*, to *Very True or Often True (2)*.

Because the normalized t-scores estimated from these two CBCL forms differ in whether child sex was adjusted (the preschool form does not normalize by sex, whereas the school aged form does), we computed the raw score for the total problems scale as the primary outcome of interest and adjusted for the version of form (preschool vs. school age), as well as child age (in years), and study site region in the analyses.

As a follow-up examination of clinically meaningful indicators of child mental health, children were classified into dichotomous categories of evidencing behaviors in the borderline and/or clinical range of total problems when their normalized t-scores (across the two form versions) were equal to or above the borderline (84th percentile) and clinical (90th percentile) thresholds, respectively. As noted above, the school age form is sex-normed whereas the preschool form is not. Child sex was also included as a covariate in all analyses.

**Regression Model Covariate Details**

A series of three regression models included three additive sets of covariates. Model 1 included methodological confounders. Model 2 included those and confounders at the family-, parent-, and child-level. Model 3 included all covariates from Models 1 and 2, and additional variables potentially on the mechanistic path between maternal stress exposure and child mental health.

Model 1: Given the multi-site nature of the total study sample, a variable capturing site was comprised of the following categories: Memphis (CANDLE), San Francisco (TIDES), Minneapolis (TIDES), Rochester (TIDES), Seattle-TIDES, Seattle-GAPPS, Yakima (GAPPS). In addition, the Child Behavior Checklist (CBCL) form type was included as a categorical covariate.

Model 2: Added covariates included a measure of family income, adjusted for household size, region of the country, and inflation. Covaried maternal factors were age, parity (number of previous pregnancies), pre-pregnancy body mass index, level of education (Less than high school, High school diploma or GED, Vocational or Technical school after high school, some College [no degree] or Associate Degree, College Graduate or Baccalaureate degree, Masters Degree, Doctoral-level/professional degree). Covaried child factors were age at outcome, year of birth, sex, and race/ethnicity (non-Hispanic White, non-Hispanic Black/African American, non-Hispanic other (incl. Asian, Native Hawaiian/Other Pacific Islander, American Indian/Alaska Native, other, multiple race), and Hispanic ethnicity (Hispanic/Latinx, any race).

Model 3: Additional covariates added in Model 3 were maternal cigarette smoking during pregnancy, child gestational age at birth, whether the child was breastfed, and maternal self-reported depression at the child age 4-6 visit. In addition to being a potential mediator of the tested associations, maternal depression is a possible source of bias in reporting of child mental health (though see [2]). Maternal depression was assessed with two different measures across the three cohorts and harmonized via the PROSetta Stone crosswalk tables (CITE). Specifically, the TIDES and GAPPS assessed maternal depression with the Patient Reported Outcome Measurement Information System (PROMIS) Depression Short Form (PROMIS-D-8), which was developed by the National Institutes of Health to measure patient-reported depression symptoms relevant to a range of chronic diseases [3,4]. This 8-item measure inquires about the frequency of depression symptoms (e.g., sadness, worthlessness, anhedonia) in the previous week; sum scores are calculates across all items and converted into a t-score. In the CANDLE cohort, maternal depression was assessed with the Center for Epidemiologic Studies Depression Scale (CES-D; CITE),which has been linked to the PROMIS-D-8 via empirical work [5]. A crosswalk table has been developed to harmonize these two measures, converting a BSI-depression subscale score to a PROMIS-D-8 t-score.

**Supplemental References**

[1] Achenbach TM. Child Behavior Checklist. In: Kreutzer JS, DeLuca J, Caplan B, editors. Encycl. Clin. Neuropsychol., New York, NY: Springer New York; 2011, p. 546–52. https://doi.org/10.1007/978-0-387-79948-3_1529.

[2] Olino TM, Michelini G, Mennies RJ, Kotov R, Klein DN. Does maternal psychopathology bias reports of offspring symptoms? A study using moderated non-linear factor analysis. J Child Psychol Psychiatry 2021;n/a. https://doi.org/10.1111/jcpp.13394.

[3] Cella D, Riley W, Stone A, Rothrock N, Reeve B, Yount S, et al. The Patient-Reported Outcomes Measurement Information System (PROMIS) developed and tested its first wave of adult self-reported health outcome item banks: 2005–2008. J Clin Epidemiol 2010;63:1179–94. https://doi.org/10.1016/j.jclinepi.2010.04.011.

[4] Teresi JA, Ocepek-Welikson K, Kleinman M, Eimicke JP, Crane K, Jones RN, et al. Analysis of Differential Item Functioning in the Depression Item Bank from the Patient Reported Outcome Measurement Information System (PROMIS): An Item Response Theory Approach 2010:35.

[5] Choi SW, Schalet B, Cook KF, Cella D. Establishing a common metric for depressive symptoms: linking the BDI-II, CES-D, and PHQ-9 to PROMIS depression. Psychol Assess 2014;26:513–27. https://doi.org/10.1037/a0035768.

**Supplemental Tables**

**Supplemental Table 1. Pearson correlations among key study variables**

**
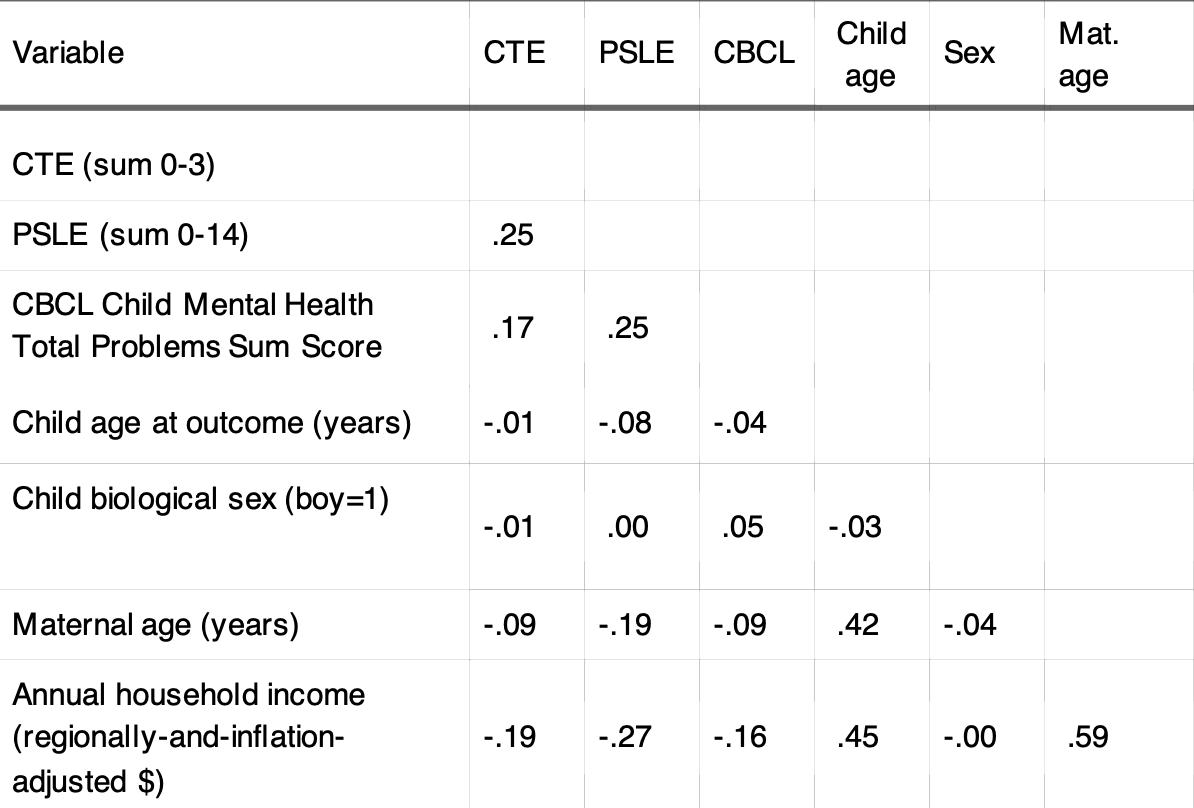
**

*Notes*. CTE = childhood traumatic events; PSLE = pregnancy stressful life events; CBCL = Child Mental Health Total Problems Sum Score; Annual Income is regionally-and-inflation-adjusted

**Supplemental Table 2**. **Unstandardized Coefficients from Extended Regression Model including Sex*Exposure Predicting Child CBCL Total Problems.**

|  | MICE  Exposures X biological sex | Complete Cases  Exposures X biological sex |
| --- | --- | --- |
| (Intercept) | 19.78 *** | 19.78 *** |
|  | (2.61) | (3.14) |
| CTE (sum 0-3) | 1.60 * | 1.05 |
|  | (0.74) | (0.78) |
| PSLE (sum 0-14) | 1.35 ** | 1.39 *** |
|  | (0.46) | (0.37) |
| sex*CTE (sum 0-3) | 0.39 | 0.60 |
|  | (1.07) | (1.09) |
| sex*PSLE (sum 0-14) | 0.55 | 0.89 + |
|  | (0.56) | (0.50) |
| CBCL Form (age 6-18) | 3.14 | 4.26 |
|  | (2.62) | (2.82) |
| site: SF | -4.83 | -4.72 |
|  | (3.79) | (4.06) |
| site: UMN | -0.46 | -0.13 |
|  | (3.63) | (3.87) |
| site: Rochester | 0.60 | 1.07 |
|  | (3.69) | (4.02) |
| site: Seattle (TIDES) | 2.48 | 3.23 |
|  | (3.76) | (4.11) |
| site: Seattle (GAPPS) | -1.30 | -2.79 |
|  | (3.12) | (3.39) |
| site: Yakima | -3.02 | -2.12 |
|  | (3.23) | (3.50) |
| year_birth2008 | -1.01 | -1.99 |
|  | (2.39) | (3.04) |
| year_birth2009 | -2.63 | -3.03 |
|  | (2.42) | (3.00) |
| year_birth2010 | -6.39 ** | -7.61 * |
|  | (2.37) | (2.96) |
| year_birth2011 | -4.79 * | -4.86 |
|  | (2.42) | (2.97) |
| year_birth2012 | -3.98 | -4.10 |
|  | (2.92) | (3.45) |
| year_birth2013 | -1.92 | -2.33 |
|  | (3.45) | (3.95) |
| year_birth2014 | -2.96 | -3.20 |
|  | (4.11) | (4.56) |
| year_birth2015 | -11.64 | -9.91 |
|  | (9.30) | (16.79) |
| Gravidity (# preg.) | -0.25 | -0.25 |
|  | (0.29) | (0.31) |
| (c)Maternal BMI | 0.04 | 0.12 + |
|  | (0.06) | (0.06) |
| (c)Maternal Age (yr) | 0.09 | 0.22 * |
|  | (0.10) | (0.10) |
| Edu: <HS | 4.09 + | 9.47 ** |
|  | (2.46) | (2.90) |
| Edu: HS or GED | 2.38 + | 1.97 |
|  | (1.31) | (1.45) |
| Edu: Vocational or Technical School | 2.05 | 1.86 |
|  | (1.32) | (1.41) |
| Edu: Grad or Professional degree | 1.63 | 1.22 |
|  | (1.12) | (1.17) |
| (c)log(Income) @hhsize 2 | 3.05 | 1.98 |
|  | (2.46) | (2.68) |
| (c)log(Income) @hhsize 3 | 0.08 | 0.98 |
|  | (1.11) | (1.04) |
| (c)log(Income) @hhsize 4 | -0.70 | -0.47 |
|  | (0.83) | (0.88) |
| (c)log(Income) @hhsize 5 | -2.52 ** | -2.76 ** |
|  | (0.93) | (0.95) |
| (c)log(Income) @hhsize 6+ | -2.36 * | -2.23 * |
|  | (1.07) | (1.12) |
| hhsize 2 | 1.73 | 1.37 |
|  | (2.50) | (2.77) |
| hhsize 3 | -0.25 | -0.70 |
|  | (1.17) | (1.26) |
| hhsize 5 | 0.56 | 0.80 |
|  | (1.07) | (1.15) |
| hhsize 6+ | 0.81 | -0.38 |
|  | (1.33) | (1.43) |
| (c)Child Age (yr) | -1.43 | -2.47 + |
|  | (1.15) | (1.31) |
| Boy | 0.97 | 0.51 |
|  | (1.16) | (1.18) |
| race_ethWhite (alone; non-Hispanic) | 4.50 *** | 4.07 ** |
|  | (1.27) | (1.39) |
| race_ethOther (and multiple race; non-Hispanic) | 2.67 | 1.09 |
|  | (1.64) | (1.78) |
| race_ethHispanic (any race) | 2.27 | 2.61 |
|  | (1.72) | (1.84) |
| Prenatal Smoking Status | 3.83 * | 3.23 |
|  | (1.76) | (2.02) |
| (c)Gestational Age (wks) | -0.30 | -0.41 |
|  | (0.27) | (0.29) |
| (c)Birthweight (kg) | -0.43 | 0.36 |
|  | (0.94) | (1.01) |
| Breastfeeding | -2.03 + | -1.47 |
|  | (1.09) | (1.25) |
| (c)Maternal Depression | 0.58 *** | 0.55 *** |
|  | (0.06) | (0.06) |
| *N* | 1948 | 1484 |
| *R2* | 0.18 | 0.20 |
| *** *p* < 0.001; ** *p* < 0.01; * *p* < 0.05; + *p* < 0.1. | | |

**​​**

**Supplemental Table 3. Unstandardized Coefficients from Regression Models 2 and 3 Predicting Child CBCL Total Problem for the Pooled-Imputed Sample and Cohort-specific Samples**

|  | MI-all  Model 2 | MI-all  Model 3 | CANDLE  Model 2 | CANDLE  Model 3 | GAPPS  Model 2 | GAPPS  Model 3 | TIDES  Model 2 | TIDES  Model 3 | complete data Model 2 | complete data Model 3 |
| --- | --- | --- | --- | --- | --- | --- | --- | --- | --- | --- |
| (Intercept) | 18.28 *** | 19.17 *** | 19.98 *** | 20.96 *** | 5.64 | 6.74 | 10.13 ** | 13.77 ** | 17.74 *** | 18.60 *** |
|  | (2.56) | (2.54) | (3.25) | (3.17) | (7.53) | (8.01) | (3.89) | (4.65) | (3.10) | (3.08) |
| CTE (sum 0-3) | 2.47 *** | 1.79 ** | 2.18 ** | 1.45 + | 2.40 * | 1.85 | 2.90 ** | 2.33 * | 1.96 *** | 1.33 * |
|  | (0.56) | (0.55) | (0.80) | (0.78) | (1.19) | (1.20) | (0.96) | (0.95) | (0.58) | (0.57) |
| PSLE (sum 0-14) | 2.12 *** | 1.64 *** | 1.69 *** | 1.17 ** | 2.65 *** | 2.52 *** | 2.51 *** | 1.88 *** | 2.27 *** | 1.87 *** |
|  | (0.30) | (0.31) | (0.42) | (0.42) | (0.63) | (0.64) | (0.51) | (0.52) | (0.26) | (0.26) |
| CBCL Form (age 6-18) | 3.48 | 3.23 |  |  | 3.67 | 4.01 |  |  | 4.27 | 4.41 |
|  | (2.69) | (2.62) |  |  | (3.55) | (3.53) |  |  | (2.91) | (2.82) |
| site: SF | -6.65 + | -4.91 |  |  |  |  |  |  | -5.97 | -4.79 |
|  | (3.90) | (3.79) |  |  |  |  |  |  | (4.17) | (4.06) |
| site: UMN | -2.57 | -0.57 |  |  |  |  | 3.21 | 3.38 + | -1.60 | -0.24 |
|  | (3.72) | (3.63) |  |  |  |  | (2.00) | (1.97) | (3.98) | (3.87) |
| site: Rochester | -1.49 | 0.46 |  |  |  |  | 4.92 * | 4.96 * | 0.06 | 1.01 |
|  | (3.79) | (3.69) |  |  |  |  | (2.48) | (2.47) | (4.13) | (4.02) |
| site: Seattle (TIDES) | -0.10 | 2.37 |  |  |  |  | 6.43 ** | 6.77 ** | 1.27 | 3.16 |
|  | (3.86) | (3.76) |  |  |  |  | (2.12) | (2.09) | (4.21) | (4.11) |
| site: Seattle (GAPPS) | -0.68 | -1.33 |  |  |  |  |  |  | -2.05 | -2.74 |
|  | (3.18) | (3.12) |  |  |  |  |  |  | (3.45) | (3.39) |
| site: Yakima | -4.75 | -3.07 |  |  | -4.31 + | -2.47 |  |  | -3.29 | -2.10 |
|  | (3.32) | (3.22) |  |  | (2.20) | (2.39) |  |  | (3.59) | (3.50) |
| year_birth2008 | -2.03 | -1.01 | -2.09 | -0.90 |  |  |  |  | -2.49 | -1.82 |
|  | (2.45) | (2.38) | (2.66) | (2.56) |  |  |  |  | (3.13) | (3.04) |
| year_birth2009 | -3.82 | -2.60 | -4.42 | -2.98 |  |  |  |  | -3.60 | -2.79 |
|  | (2.49) | (2.42) | (2.77) | (2.67) |  |  |  |  | (3.07) | (2.99) |
| year_birth2010 | -7.08 ** | -6.36 ** | -7.50 ** | -6.65 * |  |  |  |  | -7.80 * | -7.40 * |
|  | (2.44) | (2.37) | (2.74) | (2.63) |  |  |  |  | (3.04) | (2.96) |
| year_birth2011 | -5.67 * | -4.81 * | -6.15 * | -5.04 + |  |  |  |  | -5.25 + | -4.73 |
|  | (2.49) | (2.42) | (2.76) | (2.67) |  |  |  |  | (3.04) | (2.97) |
| year_birth2012 | -4.33 | -4.05 |  |  | 2.42 | 2.41 | 2.47 | 2.09 | -4.13 | -4.09 |
|  | (2.99) | (2.92) |  |  | (3.27) | (3.30) | (1.73) | (1.71) | (3.54) | (3.45) |
| year_birth2013 | -2.50 | -1.88 |  |  | 5.79 | 5.88 | 2.15 | 2.08 | -2.58 | -2.16 |
|  | (3.53) | (3.45) |  |  | (4.09) | (4.18) | (3.05) | (3.02) | (4.05) | (3.95) |
| year_birth2014 | -3.19 | -2.91 |  |  | 5.24 | 5.45 |  |  | -3.19 | -3.00 |
|  | (4.19) | (4.11) |  |  | (4.50) | (4.64) |  |  | (4.67) | (4.56) |
| year_birth2015 | -15.60 + | -12.09 |  |  | -8.55 | -6.49 |  |  | -8.37 | -10.77 |
|  | (9.47) | (9.27) |  |  | (9.33) | (9.61) |  |  | (17.24) | (16.80) |
| Gravidity (# preg.) | -0.26 | -0.25 | -0.46 | -0.36 | -0.11 | -0.26 | -0.51 | -0.45 | -0.24 | -0.24 |
|  | (0.30) | (0.29) | (0.49) | (0.47) | (0.51) | (0.52) | (0.57) | (0.57) | (0.32) | (0.31) |
| (c)Maternal BMI | 0.07 | 0.05 | 0.01 | -0.01 | 0.12 | 0.11 | 0.24 + | 0.20 | 0.15 * | 0.12 + |
|  | (0.06) | (0.06) | (0.08) | (0.07) | (0.13) | (0.13) | (0.13) | (0.13) | (0.06) | (0.06) |
| (c)Maternal Age (yr) | 0.10 | 0.09 | 0.05 | 0.03 | 0.18 | 0.20 | 0.13 | 0.09 | 0.24 * | 0.22 * |
|  | (0.10) | (0.10) | (0.15) | (0.15) | (0.18) | (0.18) | (0.19) | (0.19) | (0.10) | (0.10) |
| Edu: <HS | 5.66 * | 4.10 + | 4.35 | 2.93 | 30.06 ** | 27.36 ** | 4.18 | 1.40 | 11.13 *** | 9.47 ** |
|  | (2.54) | (2.46) | (3.19) | (3.04) | (10.25) | (10.32) | (5.07) | (5.25) | (2.95) | (2.91) |
| Edu: HS or GED | 2.57 + | 2.43 + | 3.97 * | 3.74 * | 1.41 | 1.45 | -5.00 | -5.73 | 1.99 | 2.11 |
|  | (1.36) | (1.31) | (1.68) | (1.62) | (3.83) | (3.85) | (3.79) | (3.78) | (1.48) | (1.45) |
| Edu: Vocational or Technical School | 1.97 | 2.02 | 5.25 * | 4.39 * | -0.13 | 0.31 | -2.73 | -2.82 | 1.91 | 1.82 |
|  | (1.35) | (1.31) | (2.09) | (1.99) | (2.46) | (2.47) | (2.63) | (2.61) | (1.45) | (1.41) |
| Edu: Grad or Professional degree | 1.47 | 1.65 | 1.68 | 3.06 + | 3.11 | 2.68 | 0.83 | 0.58 | 1.09 | 1.29 |
|  | (1.15) | (1.11) | (1.91) | (1.85) | (2.42) | (2.44) | (1.73) | (1.70) | (1.21) | (1.17) |
| (c)log(Income) @hhsize 2 | 1.48 | 2.99 | 2.07 | 3.29 | -6.82 | -5.19 | 7.48 | 8.47 + | -0.36 | 1.84 |
|  | (2.50) | (2.46) | (3.54) | (3.47) | (4.75) | (4.72) | (5.11) | (5.07) | (2.75) | (2.68) |
| (c)log(Income) @hhsize 3 | -0.51 | 0.09 | 0.99 | 0.87 | -3.64 | -3.70 | -3.97 + | -2.82 | 0.40 | 1.00 |
|  | (1.15) | (1.11) | (1.48) | (1.41) | (3.88) | (3.84) | (2.29) | (2.26) | (1.07) | (1.04) |
| (c)log(Income) @hhsize 4 | -1.75 * | -0.70 | -1.43 | -0.43 | -2.48 | -0.85 | -3.74 * | -2.83 + | -1.42 | -0.46 |
|  | (0.84) | (0.83) | (1.14) | (1.10) | (2.84) | (3.00) | (1.66) | (1.66) | (0.90) | (0.88) |
| (c)log(Income) @hhsize 5 | -3.75 *** | -2.55 ** | -3.50 ** | -1.94 | 0.59 | 1.36 | -4.04 + | -3.39 | -3.89 *** | -2.81 ** |
|  | (0.95) | (0.93) | (1.28) | (1.24) | (2.74) | (2.74) | (2.10) | (2.11) | (0.97) | (0.95) |
| (c)log(Income) @hhsize 6+ | -3.35 ** | -2.36 * | -3.07 * | -1.20 | 1.45 | 2.00 | -4.59 | -4.57 | -2.89 * | -2.21 * |
|  | (1.10) | (1.06) | (1.45) | (1.40) | (3.73) | (3.72) | (3.02) | (3.06) | (1.16) | (1.13) |
| hhsize 2 | 1.53 | 1.67 | -2.13 | -1.75 | 1.30 | 2.13 | 7.17 | 6.81 | 0.29 | 1.13 |
|  | (2.55) | (2.49) | (2.85) | (2.74) | (7.25) | (7.22) | (6.32) | (6.21) | (2.85) | (2.77) |
| hhsize 3 | -0.45 | -0.22 | -1.14 | -1.17 | 1.95 | 1.64 | -1.44 | -0.75 | -0.99 | -0.62 |
|  | (1.20) | (1.17) | (1.80) | (1.72) | (3.13) | (3.20) | (1.94) | (1.93) | (1.29) | (1.26) |
| hhsize 5 | 0.79 | 0.60 | 3.29 * | 2.75 + | -0.02 | -0.18 | -2.89 | -2.81 | 0.85 | 0.90 |
|  | (1.09) | (1.07) | (1.59) | (1.53) | (2.51) | (2.51) | (2.00) | (1.98) | (1.18) | (1.15) |
| hhsize 6+ | 0.78 | 0.87 | 3.21 + | 3.85 * | -0.10 | -0.27 | -2.04 | -2.16 | -0.45 | -0.30 |
|  | (1.37) | (1.33) | (1.90) | (1.83) | (2.90) | (2.90) | (2.70) | (2.66) | (1.47) | (1.43) |
| (c)Child Age (yr) | -0.43 | -1.39 | -1.43 | -2.70 | 0.58 | -0.37 | 1.25 | 1.00 | -1.62 | -2.43 + |
|  | (1.19) | (1.15) | (1.75) | (1.69) | (2.30) | (2.33) | (2.33) | (2.30) | (1.34) | (1.31) |
| Boy | 1.93 * | 2.01 * | 1.45 | 1.11 | 0.60 | 1.27 | 3.21 * | 3.25 * | 2.09 * | 2.16 * |
|  | (0.80) | (0.79) | (1.16) | (1.13) | (1.80) | (1.85) | (1.41) | (1.42) | (0.88) | (0.87) |
| race_ethWhite (alone; non-Hispanic) | 4.35 *** | 4.57 *** | 3.78 * | 4.36 ** | 6.59 | 7.71 | 4.58 | 3.60 | 4.21 ** | 4.11 ** |
|  | (1.28) | (1.27) | (1.65) | (1.64) | (6.40) | (6.41) | (2.98) | (2.99) | (1.39) | (1.39) |
| race_ethOther (and multiple race; non-Hispanic) | 2.54 | 2.68 | 2.42 | 2.20 | 8.12 | 9.16 | 0.54 | -0.22 | 1.32 | 1.06 |
|  | (1.68) | (1.64) | (2.74) | (2.64) | (6.42) | (6.44) | (3.46) | (3.44) | (1.81) | (1.78) |
| race_ethHispanic (any race) | 1.26 | 2.24 | 1.88 | 1.91 | 4.41 | 6.27 | 0.19 | -0.07 | 1.82 | 2.51 |
|  | (1.76) | (1.72) | (3.44) | (3.30) | (6.50) | (6.55) | (3.26) | (3.21) | (1.88) | (1.85) |
| Prenatal Smoking Status |  | 3.67 * |  | 3.61 + |  | 5.17 |  | 3.88 |  | 2.91 |
|  |  | (1.75) |  | (2.12) |  | (5.78) |  | (4.18) |  | (2.01) |
| (c)Gestational Age (wks) |  | -0.30 |  | -0.23 |  | -0.15 |  | -0.46 |  | -0.42 |
|  |  | (0.26) |  | (0.41) |  | (0.56) |  | (0.47) |  | (0.29) |
| (c)Birthweight (kg) |  | -0.40 |  | -0.35 |  | -1.00 |  | 0.27 |  | 0.44 |
|  |  | (0.93) |  | (1.41) |  | (2.10) |  | (1.59) |  | (1.01) |
| Breastfeeding |  | -1.98 + |  | -1.97 |  | -3.18 |  | -1.98 |  | -1.40 |
|  |  | (1.09) |  | (1.31) |  | (3.76) |  | (2.87) |  | (1.25) |
| (c)Maternal Depression |  | 0.58 *** |  | 0.80 *** |  | 0.31 * |  | 0.42 *** |  | 0.55 *** |
|  |  | (0.06) |  | (0.09) |  | (0.12) |  | (0.09) |  | (0.06) |
| N | 1948 | 1948 | 1030 | 1030 | 377 | 377 | 541 | 541 | 1484 | 1484 |
| R2 | 0.13 | 0.18 | 0.11 | 0.19 | 0.20 | 0.23 | 0.19 | 0.23 | 0.14 | 0.19 |

*Notes.* Reference categories: Site = CANDLE/Memphis; Race/Ethnicity=Black, non-Hispanic*;* CBCL form = 1.5-5*;* Child sex=girl*;* Highest level of education=College degree*;* household size=4*;* (c)=variable was mean-centered for model interpretation.

**Supplemental Figure 1. Illustration of Standardized Coefficients from Regression Models, single-cohort samples.**


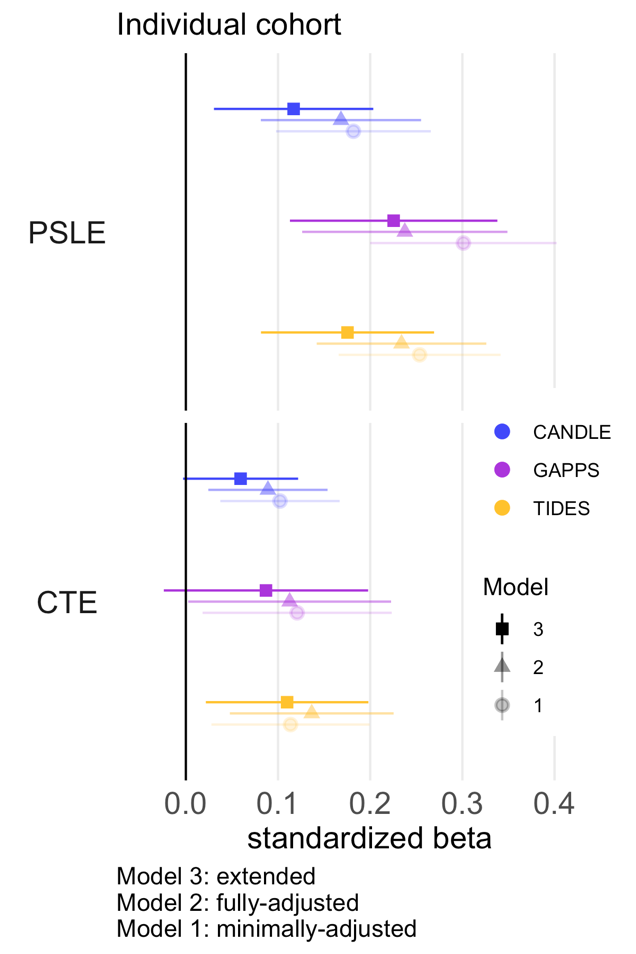

Supplement: Supplementary file 1 — Supplementary file1 (DOCX 257 KB) [file 127_2022_2401_MOESM1_ESM.docx]
